# Balloon vs. balloon—comparison of hemolysis and renal markers after cryoballoon vs. ballon-in-basket pulsed field pulmonary vein isolation

**DOI:** 10.3389/fcvm.2025.1681098

**Published:** 2025-09-16

**Authors:** Jan-Per Wenzel, Raed Abdessadok, Sascha Hatahet, Charlotte Eitel, Julius Nikorowitsch, Roman Mamaev, Sorin Popescu, Samuel Reincke, Anna Traub, Behnam Subin, Suzanne de Waha, Tanja Zeller, Karl-Heinz Kuck, Roland Richard Tilz

**Affiliations:** ^1^ Department of Rhythmology, University Heart Center Lübeck, University Hospital Schleswig-Holstein, Lübeck, Germany; ^2^ German Center for Cardiovascular Research (DZHK), Partner Site Lübeck, Lübeck, Germany; ^3^ Institute for Cardiogenetics, University Heart Center Lübeck, University Hospital Schleswig-Holstein, Lübeck, Germany

**Keywords:** balloon catheter, cryoablation, pulsed field ablation, hemolysis, renal function

## Abstract

**Background and aims:**

Single-shot ablation systems are widely used for pulmonary vein isolation (PVI) in atrial fibrillation (AF). Cryoballoon ablation (CBA) ablation is a well-established thermal method, while balloon-in-basket pulsed field ablation (BiB-PFA) represents a novel non-thermal modality. Both may elicit systemic effects, including hemolysis and renal stress. This study aimed to compare biomarker changes indicative of hemolysis and renal function following CBA vs. BiB-PFA.

**Methods:**

In this prospective, non-randomized, single-center study, patients undergoing first-time PVI with either CBA or BiB-PFA were enrolled. Venous blood samples were collected before PVI and at day 1 post procedure. Laboratory analyses included lactate dehydrogenase (LDH), haptoglobin, hemoglobin, myoglobin, total bilirubin, creatinine, and estimated glomerular filtration rate (eGFR).

**Results:**

A total of 100 patients were included (CBA: *n* = 50; BiB-PFA: *n* = 50). Acute and first-pass PVI was achieved in all cases. CBA resulted in a significantly greater increase in LDH (Δ+60 vs. + 47 U/L; *p* = 0.038) and a more pronounced decline in haptoglobin (Δ−13 vs. −3 mg/dl; *p* = 0.003). Hemoglobin decreased after BiB-PFA (Δ−0.62 g/dl) but slightly increased after CBA (Δ+0.18 g/dl; *p* < 0.001). Myoglobin and bilirubin changes were comparable. There was no significant difference in kidney function change between the groups (CBA: Δ−2.0 vs. BiB-PFA: glomerular filtration rate −1.0 ml/min; *p* = 0.522).

**Conclusion:**

While CBA was associated with more pronounced hematologic changes, kidney function did not differ between groups. These findings emphasize the systemic effects of catheter design and energy modality while supporting the renal safety of both techniques.

## Graphical Abstract

**Figure:**
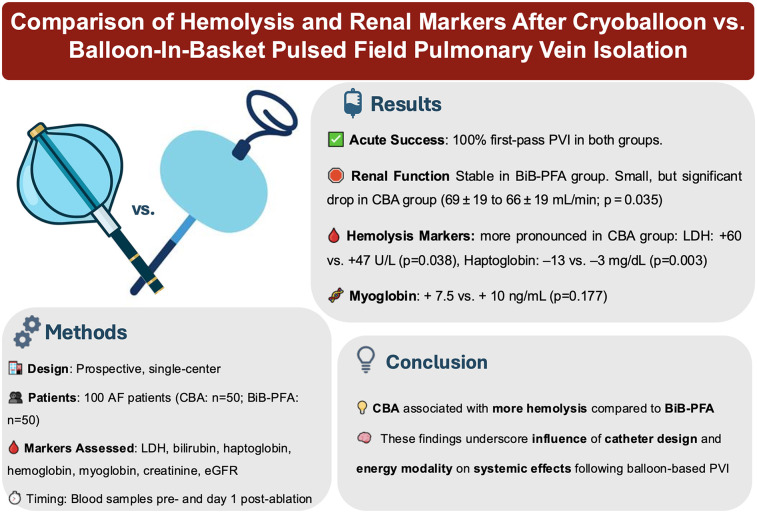

## Introduction

Pulmonary vein isolation (PVI) remains the cornerstone of catheter ablation for atrial fibrillation (AF). Single-shot ablation systems have gained widespread adoption due to their procedural efficiency and reproducibility. Among these, cryoballoon ablation (CBA) represents an established thermal technique with a well-characterized safety profile and consistent clinical outcomes.[Fig F1][Fig F2]

Pulsed field ablation (PFA), a non-thermal modality utilizing irreversible electroporation, has recently emerged as a promising alternative for PVI (1). PFA selectively targets myocardial tissue while sparing adjacent structures, potentially reducing the risk of thermal injury. While initial studies have demonstrated favorable efficacy and safety, concerns about systemic effects—particularly hemolysis and renal dysfunction—have been raised, especially with high-energy, multi-application PFA protocols (2–4).

The balloon-in-basket (BiB) PFA system introduces a novel design combining a semi-compliant balloon with spline-mounted electrodes, enabling circumferential energy delivery with contact-modulated dose control. This catheter concept differs fundamentally from existing thermal and PFA platforms, including the widely used pentaspline PFA catheter, and may reduce blood–electrode interface exposure (5, 6).

CBA is an established procedure with a distinct complication profile. Hemolysis is observed rarely, and renal impairment has thus far been reported only in isolated cases (7, 8). A direct head-to-head functional comparison of hemolysis and renal outcomes between CBA and the novel BiB-PFA system has not yet been conducted. Hemolysis and renal impairment, while infrequent, represent clinically relevant complications that may be influenced by catheter geometry, energy modality, and procedural parameters.

This study aimed to compare hemolytic and renal biomarker changes after CBA vs. BiB-PFA and to determine whether catheter-specific factors contribute to differential systemic effects.

## Methods

### Study population and trial design

Between January 2024 and April 2025, consecutive patients with symptomatic paroxysmal or persistent AF undergoing first-time PVI using either CBA or BiB-PFA were prospectively enrolled from the Lübeck Ablation Registry. Twenty-seven of the 50 patients BiB-PFA patients also participated in the Abbott-sponsored CE Mark study. The study protocol was approved by the institutional ethics committee (Lübeck Ablation Registry, approval number WF-028/15), and all participants provided written informed consent. The study adhered to the Declaration of Helsinki principles. Inclusion criteria were age ≥18 years and documented symptomatic AF. Patients with known hematologic disorders predisposing to hemolysis and hepatic dysfunction with elevated bilirubin levels were excluded.

### General procedural management

All patients underwent standardized preprocedural evaluation according to institutional protocols. In patients with elevated thromboembolic risk, transesophageal echocardiography was performed to exclude left atrial thrombus. Anticoagulation was managed based on medication type: for patients on vitamin K antagonists, ablation was performed at a therapeutic INR (2.0–3.0); for those on direct oral anticoagulants (DOACs), the morning dose was withheld on the day of the procedure.

Procedures were performed under deep sedation using propofol, midazolam, and fentanyl. In selected cases, continuous propofol infusion was omitted to maintain partial patient responsiveness. Analgesia was supplemented with metamizole and lidocaine as needed. Vascular access was obtained via one or two femoral vein punctures under ultrasound guidance using 8-French sheaths. A diagnostic catheter was placed in the coronary sinus, and left atrial access was achieved via a single transseptal puncture (modified Brockenbrough technique) using an SL1 sheath under fluoroscopic guidance. Intravenous unfractionated heparin was administered immediately after transseptal access to maintain an activated clotting time (ACT) > 300 s.

### Cryoballoon procedure

CBA PVI was performed using either the POLARx™ FIT cryoballoon (28 or 31 mm; Boston Scientific) or the Arctic Front Advance Pro™ cryoballoon (28 mm; Medtronic), each introduced via a dedicated steerable sheath. Both CBA systems followed the same procedural protocol. For procedures using the POLARx™ FIT system, the 28 mm balloon was used by default and always in the first PV treated; the 31 mm balloon was only selected in cases of large PV ostia or if optimal occlusion could not be achieved with the 28 mm balloon. PV angiography was performed via the SL1 sheath to visualize PV anatomy and guide balloon positioning. A time-to-isolation (TTI)-guided ablation protocol was applied with a standard freeze duration of 180 s. If TTI was >60 s, one bonus application was delivered. The diagnostic catheter was repositioned from the coronary sinus to the superior vena cava for phrenic nerve pacing, avoiding the need for additional vascular access. Additional esophageal temperature monitoring was performed for safety. Acute PVI was confirmed via entrance block using a circular mapping catheter.

### Balloon-in-basket PFA procedure

BiB-PFA procedures followed the predefined CE Mark study protocol (5). In 27 patients, pre-ablation three-dimensional voltage mapping of the left atrium was conducted using a high-density mapping catheter. In subsequent cases, anatomical reconstruction was performed directly with the ablation catheter. Following completion of mapping, the transseptal sheath was exchanged for a steerable 13 Fr sheath (Agilis™ NxT, Abbott). PV access was obtained using a 0.035-inch guidewire to ensure stable catheter positioning and support. PFA energy was delivered using either nominal (1800V) or reduced (1,400 V) waveform settings. With nominal voltage, a minimum of two applications per PV were performed with catheter rotation between deliveries. At reduced voltage, three applications were typically applied. In all cases, the number of applications per PV was limited to a maximum of eight. Phrenic nerve capture was assessed for right-sided PVs by pacing through the catheter splines. If diaphragmatic stimulation was observed, ablation was conducted using a low-voltage waveform (1,400 V), typically with at least three applications. Phrenic testing was repeated after each catheter repositioning. In five patients with posterior substrate, additional posterior wall isolation of the left atrium was performed using four targeted applications at 1,800 V, with selective deactivation of splines lacking adequate wall contact. Following ablation, a standardized 20-minute observation period was observed before post-procedural voltage remapping. Phrenic nerve integrity was reconfirmed by pacing from the superior vena cava.

### Postprocedural management

Hemostasis was achieved using vascular closure devices or a figure-of-eight suture with compression. Compression bandages were removed after 1–4 hours, and sutures were removed the following day. Transthoracic echocardiography was routinely performed immediately post-ablation, after 1 hour, and again on the first postoperative day to exclude pericardial effusion. Oral anticoagulation was resumed six hours post procedure and continued for at least two months. Long-term anticoagulation was guided by CHA₂DS₂-VASc or CHA₂DS₂-VA score, according to current guidelines.

### Intravenous fluid management

During the procedure, intravenous fluid administration was employed in patients presenting with low arterial blood pressure and/or signs of low intravascular volume status. In these cases, up to 500–1,000 ml of balanced full-electrolyte solution was administered to stabilize hemodynamics.

There was no standardized postprocedural intravenous fluid regimen. However, in patients with a baseline estimated glomerular filtration rate (eGFR) < 60 ml/min/1.73 m², a single infusion of 500–1,000 ml of balanced full-electrolyte solution was administered the day after the procedure, following routine blood sampling. The use of intravenous fluids was guided by clinical judgement, individual renal function, and volume status.

### Blood sampling and analysis

Venous blood samples were obtained at two time points: (1) immediately after femoral vascular access before any ablation was performed, and (2) on the morning of the first postprocedural day. All patients were fasting at the time of blood sampling. A panel of hematologic, renal, and hemolysis-related markers was assessed, including hemoglobin, leukocyte and platelet counts, lactate dehydrogenase (LDH), total bilirubin, haptoglobin, creatinine, estimated glomerular filtration rate (eGFR), and myoglobin. Leukocyte and platelet counts were measured from EDTA whole blood via fluorescence flow cytometry (XN-9000, Sysmex). Hemoglobin was determined by visible photometry from lithium-heparinized whole blood (IL GEM4000, Instrumentation Laboratory). Plasma markers—LDH, bilirubin, creatinine, haptoglobin, eGFR—were analyzed using the Cobas pro c503 analyzer (Roche Diagnostics). Creatinine was measured enzymatically; eGFR was calculated using the CKD-EPI formula. Myoglobin was analyzed via electrochemiluminescence immunoassay (ECLIA) on the Cobas e801 platform. All laboratory testing was conducted at the central clinical chemistry laboratory of the University Hospital Schleswig-Holstein, Campus Lübeck, according to certified protocols and quality standards.

### Statistical analysis

Continuous variables were summarized either as mean ± standard deviation (SD) for normally distributed data or median with interquartile range (IQR; 25th–75th percentile) for non-normally distributed data, as determined by the Shapiro–Wilk test. Categorical variables were expressed as absolute counts and corresponding percentages. Comparisons of continuous variables within groups were performed using either the paired *t*-test or the Wilcoxon signed-rank test, depending on normality. For between-group analyses, either the unpaired *t*-test or the Mann–Whitney *U*-test was employed, depending on the underlying distributional characteristics. Normality was assessed based on the distribution of raw data for independent samples and the distribution of differences for paired samples. If non-normality was present in either comparison group, the respective non-parametric method was applied (e.g., Mann–Whitney *U*-test for between-group differences in age). Comparisons of categorical variables were conducted using Fisher's exact test. A two-sided *p*-value < 0.05 was considered indicative of statistical significance. All statistical computations were conducted using IBM SPSS Statistics for Mac, version 29.0.2.0 (IBM Corp., Armonk, NY, USA).

## Results

### Baseline characteristics

**Figure 1 F1:**
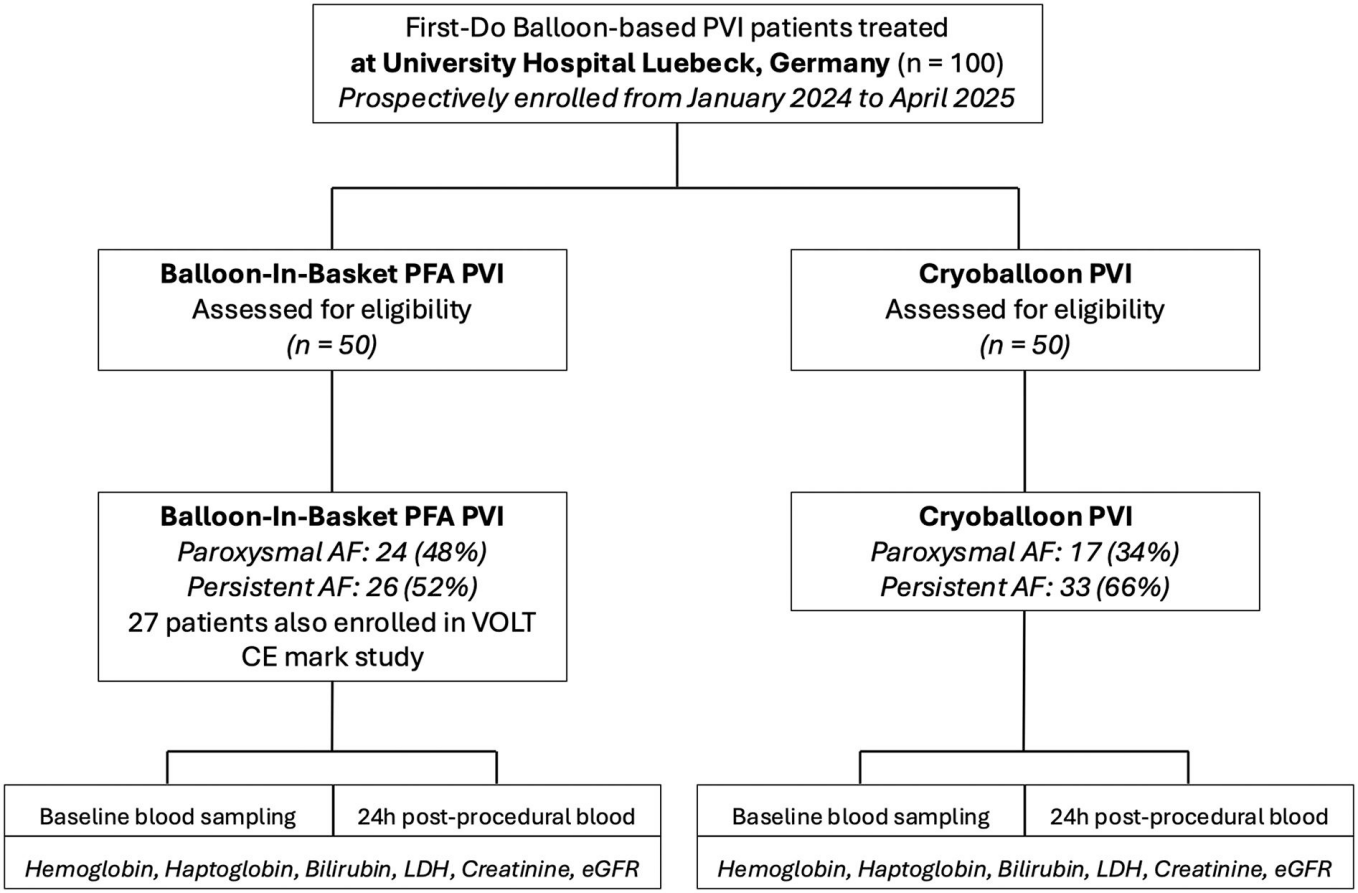
Study PRISMA (STROBE format). AF, atrial fibrillation; LDH, Lactate Dehydrogenase; PFA, pulsed field ablation; PVI, pulmonary vein isolation.

A total of 100 patients with AF were enrolled, with 50 patients each undergoing PVI using the CBA and 50 treated with the BiB-PFA system (Figure 1). Baseline characteristics are summarized in Table 1. Patients in the CBA group were significantly older than those in the BiB-PFA group [74.0 [62.75–79.0] vs. 66.0 [60.5–73.0] years; *p* = 0.01]. No significant between-group differences were observed for sex (female: 44.0% vs. 46.0%; *p* = 0.841), body mass index [28.71 [24.39–31.97] vs. 27.16 [23.70–28.73] kg/m²; *p* = 0.115], or AF type (paroxysmal: 34% vs. 48%; persistent: 66% vs. 52%; *p* = 0.097). The prevalence of cardiovascular comorbidities was comparable across groups.

**Table 1:** Baseline characteristics.

Variable	CBA (*n* = 50)	BiB-PFA (*n* = 50)	*p*-Value
Age (years)	74.0 [62.75–79.0]	66.0 [60.5–73.0]	0.01
Female sex *n* (%)	22 (44.0 %)	23 (46.0 %)	0.841
BMI (kg/m²)	28.7 [24.4–32.0]	27.2 [23.7–28.7]	0.115
AF type: paroxysmal	17 (34,0 %)	24 (48.0 %)	0.222
AF type: persistent	33 (66,0%)	26 (52.0 %)	0.222
Arterial hypertension, *n* (%)	33 (64,7%)	32 (61,5%)	0.211
Diabetes mellitus, *n* (%)	9 (18,0%)	7 (14.0 %)	0.587
Coronary artery disease, *n* (%)	11 (22.0 %)	7 (14.0 %)	0.3
Heart failure, *n* (%)	11 (22.0 %)	7 (14.0 %)	0.3
Prior stroke or TIA, *n* (%)	3 (6.0 %)	3 (6.0 %)	1.0
OSA, *n* (%)	4 (7,8%)	3 (5,8%)	1.0
CHA₂DS₂-VASc-score	3 [2–4]	2 [1–3]	0.715

Data are presented as median [interquartile range], or absolute number (percentage), as appropriate. AF, atrial fibrillation; TIA, transient ischemic attack; OSA, obstructive sleep apnea.

There were no significant differences in CHA₂DS₂-VASc score [3.0 [2.0–4.0] vs. 2.0 [1.0–3.0]; *p* = 0.715], left atrial volume index [41.6 [35.2–49.3] vs. 37.9 [26.5–48.6] ml/m²; *p* = 0.436], or left ventricular ejection fraction [53.0 [50.0–55.0]% vs. 55.0 [54.0–60.0]%; *p* = 0.392].

### Procedural characteristics

Acute PVI was achieved in all patients (Table 2). Fluoroscopy time did not differ significantly between CBA and BiB-PFA procedures [9.16 [7.10–12.60] min vs. 8.50 [6.3–11.20] min; *p* = 0.275]. Procedure duration was significantly longer in the BiB-PFA group [65.5 [47–73] min vs. 52.0 [43–58] min; *p* = 0.003], while contrast volume was significantly lower [40 [40–50] ml vs. 60 [50–60] ml; *p* < 0.001]. The median number of applications in the BiB-PFA group was 16 [13–17], compared to 4 [4–5] freeze cycles in the CBA group, with a total freeze duration of 12 [12–15] minutes.

**Table 2:** Procedural characteristics of the study population.

Parameter	CBA (*n* = 50)	BiB-PFA (*n* = 50)	*p*-Value
Fluoroscopy time (min)	9.16 [7.10–12.60]	8.50 [6.30–11.20]	0.275
Procedure duration (min)	52.0 [43.0–58.0]	65.5 [47.0–73.0]	0.003
Contrast volume (ml)	60.0 [50.0–60.0]	40 [40–50]	<0.001
Number of cardioversions, *n*	0 [0; 1]	0 [0; 1]	1
Successful acute PVI, *n* (%)	50 (100)	50 (100)	1
First-pass PVI, *n* (%)	50 (100)	50 (100)	1
Major adverse events	0 (0)	0 (0)	1
Applications	4 [4–5] freeze cycles	16 [13–17] PFA-applications	
Total freeze duration (min)	12 [12–15]		

Procedural parameters of the study population. Values are presented as median [IQR].

### Pre- and post comparisons of laboratory parameters

Both ablation modalities resulted in significant postprocedural changes in laboratory parameters (Table 3). In the CBA group, laboratory analysis revealed significant postprocedural increases in LDH [from 176 [158–215] U/L to 243 [216–264] U/L; *p* < 0.001], myoglobin [41.50 [34–62.5] ng/ml to 54 [40.5–70.5] ng/ml; *p* < 0.001], and total bilirubin [9.7 [6.65–11.8] µmol/L to 12.7 [8.25–16.95] µmol/L; *p* < 0.001]. Haptoglobin levels declined significantly [114 [82–152] mg/dl to 98 [63–126] mg/dl; *p* < 0.001], and a modest but significant decrease in eGFR was observed (68.57 ± 18.78–65.65 ± 18.9 ml/min; *p* = 0.035). Hemoglobin showed a non-significant increase (13.04 ± 1.51–13.26 ± 1.62 g/dl; *p* = 0.065), and creatinine remained unchanged [83.10 [77–102] µmol/L to 87.5 [77.50–100.5] µmol/L; *p* = 0.585] (Table 3).

**Table 3:** Comparison of pre- and postprocedural laboratory values for hemolysis and renal funtion in CBA and BiB-PFA.

Parameter	Timepoint	CBA (*n* = 50)	*p*-Value	BiB-PFA (*n* = 50)	*p*-Value
Hemoglobin (g/dl)	Pre	13.04 ± 1.51		13.87 ± 1.43	
	Post	13.26 ± 1.62	0.065	13.20 ± 1.36	<0.001
Haptoglobin (mg/dl)	Pre	114 (82–152)		115 (74–144)	
	Post	98 (63–126)	<0.001	106 (68–140)	0.067
LDH (U/L)	Pre	176.00 (158.75–215.25)		177.50 (157.50–204.75)	
	Post	243.50 (216.25–264.25)	<0,001	236,00 (204.00–265.50)	<0,001
Myoglobin (ng/ml)	Pre	41.50 (33.75–62.50)		40.00 (31.00–50.75)	
	Post	54.00 (40.50–70.50	<0.001	49.00 (42.50–71.00)	<0.001
Total bilirubin (µmol/L)	Pre	9.70 (6.65–11.80)		9,60 (7,20–12,40)	
	Post	12.70 (8.25–16.95)	<0.001	13.15 (8.13–18.58)	<0.001
Creatinin (µmol/L)	Pre	83.10 (77.15–102.25)]		84.72 ± 18.72	
	Post	87.50 (77.50–100.50)	0.585	86.13 ± 18.15	0.353
eGFR (ml/min)	Pre	68.57 ± 18.78		78.28 ± 19.46	
	Post	65.65 ± 18.94	0.035	76.53 ± 18.77	0.360

Values are presented as mean ± standard deviation or median [interquartile range]. Statistical comparisons were performed using either the *t*-test or Wilcoxon signed-rank test, depending on data distribution.

In the BiB-PFA group, LDH levels also increased significantly [178 [158–204] U/L to 236 [204–266] U/L; *p* < 0.001], as did myoglobin [40 [31–50.75] ng/ml to 49 [42.50–71.0] ng/ml; *p* < 0.001] and total bilirubin [9.60 [7.20–12.40] µmol/L to 13.15 [8.13–18.58] µmol/L; *p* < 0.001]. Hemoglobin decreased significantly (13.87 ± 1.43–13.20 ± 1.36 g/dl; *p* < 0.001). A slight, but statistically not significant, decrease in haptoglobin was observed [115 (74–144)–106 (68–140) mg/dl; *p* = 0.067]. No significant changes were observed in creatinine (84.72 ± 18.72–86.13 ± 18.15 µmol/L; *p* = 0.353) or eGFR (78.28 ± 19.46–76.53 ± 18.77 ml/min; *p* = 0.360) (Table 3).

### Intergroup comparison of laboratory parameter changes

**Figure 2 F2:**
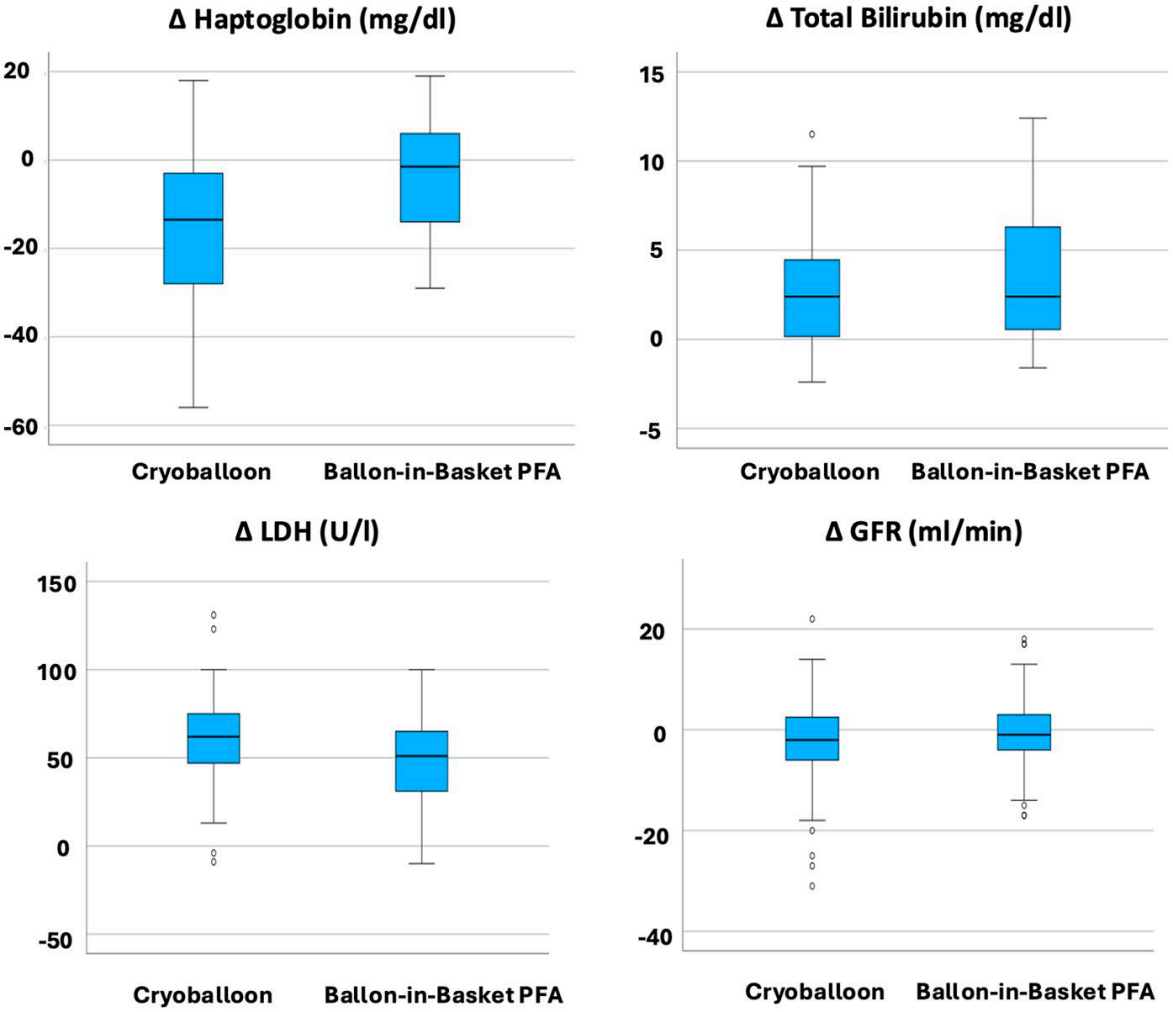
Comparison of delta values between the CBA PVI group and BiB-PFA PVI group. Box plots represent the median and interquartile range (IQR) of changes from baseline to 24 hours post-procedure. *P*-values were calculated using the Wilcoxon rank-sum test; *p* < 0.05 was considered statistically significant.

A direct comparison of postprocedural laboratory parameter changes between the CBA and the BiB-PFA group revealed several significant differences (Figure 2; Table 4). Hemoglobin levels showed a mild increase in the CBA group (Δ + 0.18 ± 0.78/dl), whereas the BiB-PFA group experienced a significant decrease (Δ −0.62 ± 1.01 g/dl; *p* < 0.001). Haptoglobin levels declined more markedly after CBA compared to the BiB-PFA group [Δ −13 [−28; −3] mg/dl vs. −3 [−14; 6], *p* = 0.003]. LDH levels increased significantly in both groups, but the rise was more pronounced in the CBA group (Δ + 60.20 ± 29.06 U/L vs. + 47.35 ± 27.91 U/L; *p* = 0.038).

**Table 4:** Comparison of periprocedural changes (delta values) in hemolysis and renal function markers between the CBA and the BiB-PFA group.

Marker	CBA	BiB-PFA	*p*-value
Δ hemoglobin (g/dl)	0.18 ± 0.78	−0.62 ± 1.01	<0.001
Δ haptoglobin (mg/dl)	−13.5 [−28; −30]	−3 [−14; 6]	0.003
Δ LDH (U/L)	60.20 ± 29.06	47.35 ± 27.91	0.038
Δ myoglobin (µg/L)	8.0 [−2.0; 15.0]	11.0 [2.0; 27.0]	0.182
Δ total bilirubin (µmol/L)	2.4 [0.0; 4.7]	2.7 [0.7; 7.4]	0.233
Δ creatinin (µmol/L)	1.82 ± 7.93	1.41 ± 10.33	0.837
Δ GFR (ml/min)	−2.0 [−6.0; 3.0]	−1.0 [−5.0; 3.0]	0.522

Values are presented as mean ± standard deviation or median [interquartile range]. Delta = postprocedural minus preprocedural value. Statistical comparisons were performed using the *t*-test or Mann–Whitney *U*-test, depending on data distribution.

No statistically significant between-group differences were observed in the changes of myoglobin [Δ + 8.0 [−2.0–15.0] µg/L vs. + 11.00 [2.00–27.0] µg/L; *p* = 0.182], total bilirubin [Δ + 2.4 [0.0; 4.7] µmol/L vs. 2.7 [0.7; 7.4] µmol/L; *p* = 0.233], creatinine (Δ + 1.82 ± 7.93 µmol/L vs. + 1.41 ± 10.33 µmol/L; *p* = 0.837), or estimated glomerular filtration rate [Δ −2.00 [−6.0–3.0] ml/min vs. −1.00 [−5.0–3.0] ml/min; *p* = 0.522].

### Correlation between number of applications and hemolysis

In the CBA group, a significant positive correlation was observed between the number of freeze cycles and the postprocedural LDH increase (*ρ* = 0.329; *p* = 0.023). A non-significant correlation was found with myoglobin (*ρ* = 0.278; *p* = 0.065). No statistically significant correlations were detected between freeze cycles and haptoglobin (*ρ* = −0.239; *p* = 0.095), hemoglobin (*ρ* = 0.024; *p* = 0.867), total bilirubin (*ρ* = 0.035; *p* = 0.814), creatinine (*ρ* = 0.116; *p* = 0.457), or estimated glomerular filtration rate (eGFR) (*ρ* = 0.007; *p* = 0.961).

In the BiB-PFA group, the number of applications showed a significant negative correlation with the decline in haptoglobin (*ρ* = −0.379; *p* = 0.023). No significant correlations were observed for LDH (*ρ* = 0.297; *p* = 0.070), hemoglobin (*ρ* = −0.164; *p* = 0.277), myoglobin (*ρ* = −0.260; *p* = 0.126), total bilirubin (*ρ* = −0.366; *p* = 0.022), creatinine (*ρ* = −0.017; *p* = 0.910), or eGFR (*ρ* = 0.055; *p* = 0.715).

## Discussion

This study presents the first direct prospective head-to-head comparison of systemic effects on hemolysis and renal impact between CBA and the novel BiB-PFA system. The main findings were:

1. CBA induced significantly greater hemolysis across all measured markers compared to BiB-PFA.
2. Both groups showed only mild and clinically irrelevant changes in renal function, with no significant difference in eGFR decline between CBA and BiB-PFA.

### Hemolysis after balloon-based ablation: energy source and interface are determinants

To date, hemolysis has not been considered a relevant complication of conventional catheter ablation techniques, such as radiofrequency (RF) or CBA (8, 9). However, with the introduction of PFA, instances of substantial intravascular hemolysis have brought this issue into sharper focus. Multiple recent investigations have consistently identified hematologic changes following PFA, particularly in systems necessitating a higher number of applications (2, 4, 10). In isolated cases, these alterations have been accompanied by acute kidney injury (11). In contrast, balloon-based PFA platforms such as the VOLT™ system typically require fewer applications, which may contribute to their more favorable systemic safety profile (6, 12).

Our findings indicate that hemolytic burden is not exclusive to PFA but also occurs with thermal balloon ablation. The extent and pattern of these changes differed substantially between the two systems. The BiB-PFA system integrates several catheter-specific features aimed at minimizing intravascular energy dispersion. Its semi-compliant balloon ensures circumferential apposition to the PV wall, while flat, laterally oriented electrodes embedded around the balloon direct the electric field toward the adjacent myocardium and away from circulating blood. Integrated contact-sensing enables real-time assessment and selective deactivation of inadequately contacting electrodes, reducing off-target energy application. The use of a biphasic waveform with lower peak voltages further optimizes tissue coupling and minimizes systemic exposure.

In contrast, the CBA system delivers cryothermal energy uniformly along its surface, which interfaces directly with circulating blood. This lack of directional control may increase erythrocyte vulnerability, explaining the observed increases in LDH and decreases in haptoglobin. Notably, haptoglobin levels in the BiB-PFA group showed a small, non-significant decrease, indicating overall stability and suggesting that BiB-PFA induces minimal hemolysis compared with the more pronounced reductions observed after CBA. The positive correlation between freeze cycles and LDH elevation supports a dose-dependent relationship; however, our data did not show consistent correlations across all hemolysis markers.

We also analyzed whether energy burden, reflected by impulse counts and freeze cycles, correlated with hemolysis. In contrast to prior studies reporting a dose-dependent relationship using a pentaspline PFA catheter, our findings showed only weak or absent correlations across most markers (13). This likely results from the strict protocol adherence in both groups, with low variability in impulse numbers—particularly in the BiB-PFA group—limiting the ability to detect subtle dose–response effects. Thus, while the amount of energy delivered can affect hemolysis, catheter design seems to play the predominant role when procedures are performed within standardized energy settings.

To our knowledge, this is the first study to show that CBA leads to more pronounced hemolytic marker changes than the BiB-PFA system. Importantly, hemolytic changes in both groups remained clinically irrelevant and were not associated with adverse outcomes.

While clinically relevant hemolysis is considered rare with CBA ablation, isolated reports suggest that specific patient-related factors can markedly increase this risk. For example, in a patient with cold agglutinin disease, cryothermal energy triggered erythrocyte agglutination and subsequent lysis, ultimately leading to hyperkalemia (7). This case highlights that, although infrequent, cryoablation may provoke hemolysis under certain immunohematologic conditions.

Overall, our findings confirm that despite procedural differences and variable application numbers, BiB-PFA is associated with a milder hemolytic profile compared with CBA, further underscoring the importance of catheter design and energy delivery characteristics for systemic safety. Of note, posterior wall isolation was performed in five patients within the BiB-PFA group. Despite the higher number of applications required, hemolytic markers remained lower than in the CBA group. This may be explained by the system's ability to selectively deactivate splines that are not in contact with atrial wall but facing toward the blood pool —thereby minimizing blood exposure even during extended ablation protocols.

Although hemolytic markers such as LDH and haptoglobin indicated only mild systemic effects in the BiB-PFA group, a statistically significant decline in hemoglobin levels was still observed. This discrepancy is likely attributable to peri-interventional fluid shifts rather than clinically relevant hemolysis, particularly in the absence of documented procedural blood loss. Given the longer procedural durations in the BiB-PFA cohort, hemodilution appears to be a plausible explanation.

### Renal function: sensitivity to hemolysis or independent stability?

Despite postprocedural hemolysis in both groups, kidney function remained largely stable. Although the CBA group showed a small but statistically significant decrease in eGFR, this change was not clinically relevant. Importantly, the difference in eGFR change between the two groups was not significant (Δ −2.0 vs. −1.0 ml/min; *p* = 0.522). Thus, our findings do not support the notion of a renal protective effect of BiB-PFA over CBA. Several confounders must be considered, including the older age and lower baseline renal function in the CBA group, as well as the higher contrast volume used. Hydration status and fluid administration may also influence renal outcomes and could contribute to the observed hemoglobin decrease in the BiB-PFA group via hemodilution.

Myoglobin increases were similar in both groups, suggesting that muscle injury was not a key factor in renal changes. Notably, BiB-PFA, compare to other PFA systems like the pentaspline PFA catheter, is associated with fewer visible muscle contractions, permitting ablation under lighter sedation—a further advantage for systemic tolerability. Collectively, these results indicate that catheter architecture, energy dispersion, and blood interface exposure are central to systemic effects. While hematologic changes were more pronounced in the CBA group, both ablation systems demonstrated a similar and acceptable renal safety profile.

### Limitations

This study has several limitations. First, its non-randomized and observational design may introduce selection bias, despite largely balanced baseline characteristics. Second, the single-center setting and moderate sample size may limit statistical power and generalizability, especially for detecting subtle or rare systemic effects. Third, postprocedural laboratory markers were assessed only once at 24 hours, potentially overlooking transient or delayed changes. Fourth, peri-interventional variables such as blood loss and fluid administration were not systematically recorded and may have contributed to observed hemoglobin changes, particularly in the BiB-PFA group.

## Conclusion

Both CBA and BiB-PFA achieved effective PVI. However, CBA was associated with more pronounced hemolysis and a slight decline in renal function. BiB-PFA showed a milder systemic profile, with a modest hemoglobin drop likely due to hemodilution. These results highlight the relevance of catheter design and energy modality for systemic safety in AF ablation.

## Data Availability

The raw data supporting the conclusions of this article will be made available by the authors, without undue reservation.
